# Magneto Twister: Magneto Deformation of the Water–Air Interface by a Superhydrophobic Magnetic Nanoparticle Layer

**DOI:** 10.1021/acs.langmuir.1c02925

**Published:** 2022-03-09

**Authors:** Udara Bimendra Gunatilake, Rafael Morales, Lourdes Basabe-Desmonts, Fernando Benito-Lopez

**Affiliations:** † Microfluidics Cluster UPV/EHU, Analytical Microsystems & Materials for Lab-on-a-Chip (AMMa-LOAC) Group, Analytical Chemistry Department, University of the Basque Country UPV/EHU, Leioa 48940, Spain; ‡ Microfluidics Cluster UPV/EHU, BIOMICs Microfluidics Group, Lascaray Research Center, University of the Basque Country UPV/EHU, Vitoria-Gasteiz 01006, Spain; § Department of Physical-Chemistry and BCMaterials, University of the Basque Country UPV/EHU, Leioa 48940, Spain; ∥ Basque Foundation of Science, IKERBASQUE, María Díaz Haroko Kalea, 3, Bilbao 48013, Spain; ⊥ Bioaraba Health Research Institute, Microfluidics Cluster UPV/EHU, Vitoria-Gasteiz 01006, Spain; # Basque Center for Materials, Applications and Nanostructures, UPV/EHU Science Park, BCMaterials, Leioa 48940, Spain

## Abstract

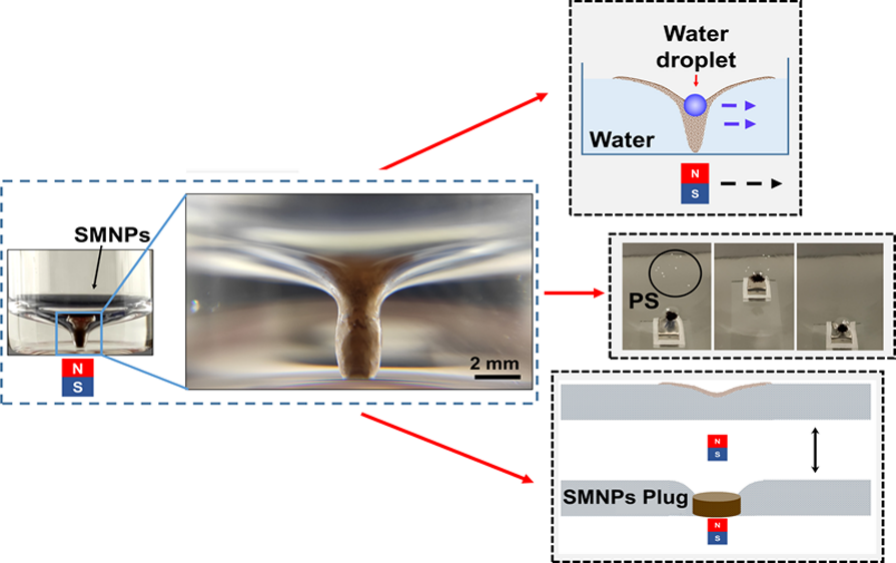

Remote manipulation of superhydrophobic surfaces provides fascinating features in water interface-related applications. A superhydrophobic magnetic nanoparticle colloid layer is able to float on the water–air interface and form a stable water–solid–air interface due to its inherent water repulsion, buoyancy, and lateral capillarity properties. Moreover, it easily bends downward under an externally applied gradient magnetic field. Thanks to that, the layer creates a stable twister-like structure with a flipped conical shape, under controlled water levels, behaving as a soft and elastic material that proportionally deforms with the applied magnetic field and then goes back to its initial state in the absence of an external force. When the tip of the twister structure touches the bottom of the water container, it provides a stable magneto movable system, which has many applications in the microfluidic field. We introduce, as a proof-of-principle, three possible implementations of this structure in real scenarios, the cargo and transport of water droplets in aqueous media, the generation of magneto controllable plugs in open surface channels, and the removal of floating microplastics from the air–water interface.

## 1 Introduction

Inspired by natural water repellent materials like lotus leaves, researchers have investigated and developed interesting superhydrophobic surfaces.^1−3^ In general, a surface with greater than 150° water contact angle is defined as superhydrophobic. According to Young’s model, to avoid wetting, the surface energy of a material should be below the surface tension of the wetting liquid.^3,4^ Moreover, the roughness of a surface affects the contact angle and hydrophobicity, which is defined by the Wenzel and Cassie-Baxter model.^5,6^ Therefore, to achieve a superhydrophobic behavior, not only the chemistry of the surface but also the surface hierarchical structure of the material is important.

The integration of magnetic properties into superhydrophobic materials or vice versa promotes remote manipulation of the material while repelling water, providing new insights for potential applications. In literature studies, magnetic phase-reinforced nanocomposites have been developed. For instance, magneto-responsive foams, which were fabricated by introducing magnetic nanoparticles (NPs) to bulk polymer matrices, were reported to remove organic contaminants from water.^7,8^ Moreover, a magnetic elastomer with a superhydrophobic surface was developed for droplet movement^9^ and to switch their dynamic wetting features.^10^ Droplets were manipulated by a local deformation of the surface of the elastomer activated by a magnetic field (MF).^9^ Recently, ferrofluid-infused laser-ablated microstructured surfaces have been introduced to manipulate gas bubbles in a programmable manner under a MF.^11^ Interestingly, a superhydrophobic magnetic microcilia array surface was recently reported to manipulate water droplets in air and oil droplets in water.^12^

On the other hand, exclusively, magnetic superhydrophobic nano-/microparticles have gained special consideration among researchers because of their easy manipulation, low remanence, and applicability at the microscale. In this regard, bare superparamagnetic Fe_3_O_4_/γ-Fe_2_O_3_ NPs and ferromagnetic Fe particles, functionalized with molecules that generate low surface energies, are directly applied, without the need to be incorporated into a bulk polymer or ceramic matrix. By using this strategy, magnetic superhydrophobic particles were used to form magnetic liquid marbles, non-adhesive droplets coated with low-surface-energy magnetic nano-/microparticles, that show extremely low friction when rolling or sliding on solid substrates.^13^ Considering the magnetic properties of these liquid marbles, small liquid droplets were manipulated by a MF.^14^ Moreover, by varying the intensity of the MF, the shell of the liquid marble can be reversibly opened and closed, enabling the removal and insertion of liquids.^13^ More recently, these magnetic marbles were used for liquid transportation,^13^ miniaturized microreactors,^14,15^ magneto-thermal reactors,^16^ and as digital microfluidics,^17,18^ among others. It is worth mentioning here that superhydrophobic magnetic NPs were used to clean oil spills or organic contaminants on water surfaces,^19−21^ wherein the superhydrophobic particles made a colloidal suspension with the oil spills due to the non-polar attraction between the oil and the outer shell of the particles but not with the water. Consequently, these magnetic colloids were easily separated from water by using a MF. Besides, Grbic et al. reported a capturing protocol of microplastics from water by binding them to hydrophobic Fe particles and subsequent recovery with a magnet.^22^ In addition, Katz et al. reversibly controlled the electrical properties of electrode interfaces using hydrophobic magnetic particles by switching the wetting properties of the electrode surface.^23^ Moreover, Meir et al. introduced the bubble marble effect, the magnetic insertion of hydrophobic metallic powders in water to keep an air bubble surrounded by a hydrophobic metallic shell and to transport solid objects within water. As a proof of concept, the underwater combustion of thermite by localized microwaves was performed.^24^ In all the above-discussed magnetic liquid marble and magnetic bubble marble investigations, the superhydrophobic magnetic particles were employed as remotely manipulable platforms.

In the present study, we investigate the deformation of a synthesized superhydrophobic and magnetic iron oxide NP (SMNP) layer–water interface. The floating SMNP layer bent downward, forming a manageable and stable water–solid–air interface under an applied MF. The layer formed a flipped conical spike (CS), similar to a storm twister, with the spike structure touching the depth of the water container using controlled water levels. This system was characterized and used for the transportation of water droplets in aqueous media, as a magnetic plug for liquid partition in open surface channels and to remove microplastics on water surfaces.

## 2 Results and Discussion

### 2.1 Magneto Deformation of the Water–Air Interface

**Figure 1:**
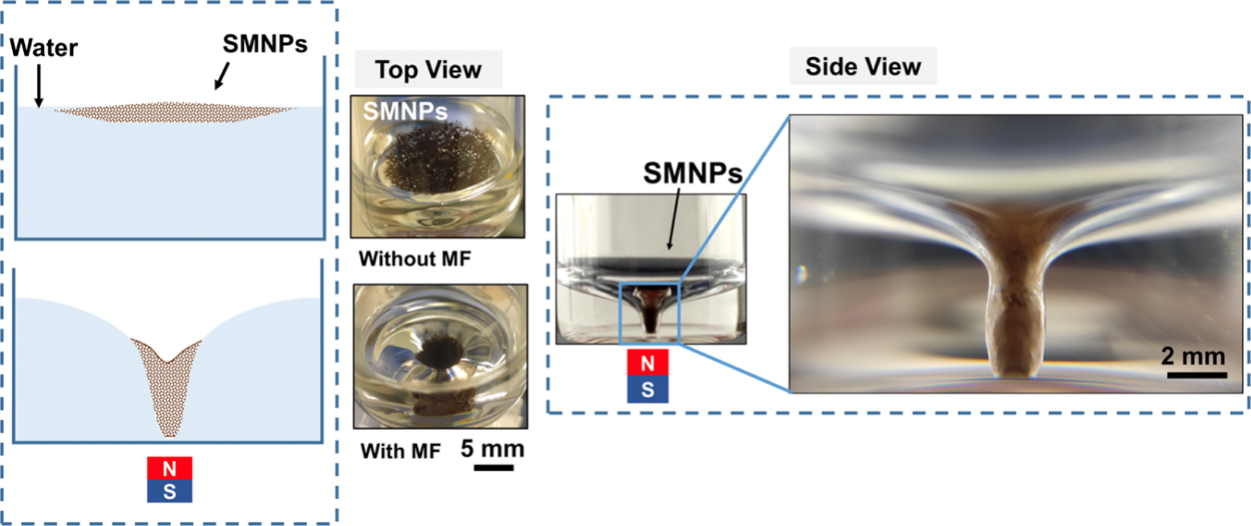
Deformation of the SMNPs layer on the water–air interface, under a MF of 42 mT. From left to right: schematic diagram of the formation of the twister, pictures of the top and side view of the magneto twister.

The synthesized low-surface-energy SMNPs were characterized by Fourier transform infrared (FTIR) spectroscopy, X-ray diffraction analysis (XRD), Raman spectroscopy, transmission electron microscopy (TEM), and using the superconducting quantum interference device (SQUID) magnetometer and goniometer, see Supporting Information, Sections S1–S3. Iron oxide magnetic NPs synthesized by the coprecipitation method can be submerged and dispersed in a bulk water medium because of their high specific gravity, high surface energy, and chemical hydrogen bond attraction between surface hydroxyls of particles and water. Nevertheless, long-chain alkyl-modified low-surface-energy SMNPs were found to float in the water–air interface, as shown in Video S6.1. The non-favorable attraction between the non-polar carbon long chain and water avoids the immersion of the particles despite the higher specific gravity. Moreover, the buoyancy and the surface tension forces also contribute to that effect, keeping the particles in the floating state on the water surface and balancing the weight of the particles.^25^ Aggregation and repulsion between the floating SMNPs are due to the induced lateral capillary forces between the particles, which cause a slight deformation of the particle–water interface (meniscus) that is related to the wetting properties of the particles.^26^ As depicted in Figure 1, these floating superhydrophobic magnetic NPs polarized under a gradient MF (*z* direction) are attracted toward the magnet by exerting a downward magnetic force *F*_m_ on the magnetic NP colloid layer floating at the air–water interface. These magnetic forces on the particles are dependent on the volume (*V*_m_) of the particles, the difference in magnetic susceptibilities (Δχ) of the medium and the magnetic particle, and the magnetic induction intensity (*B*) and its gradient (∇*B*), as shown in eq 1.^27^
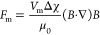
1

**Figure 2:**
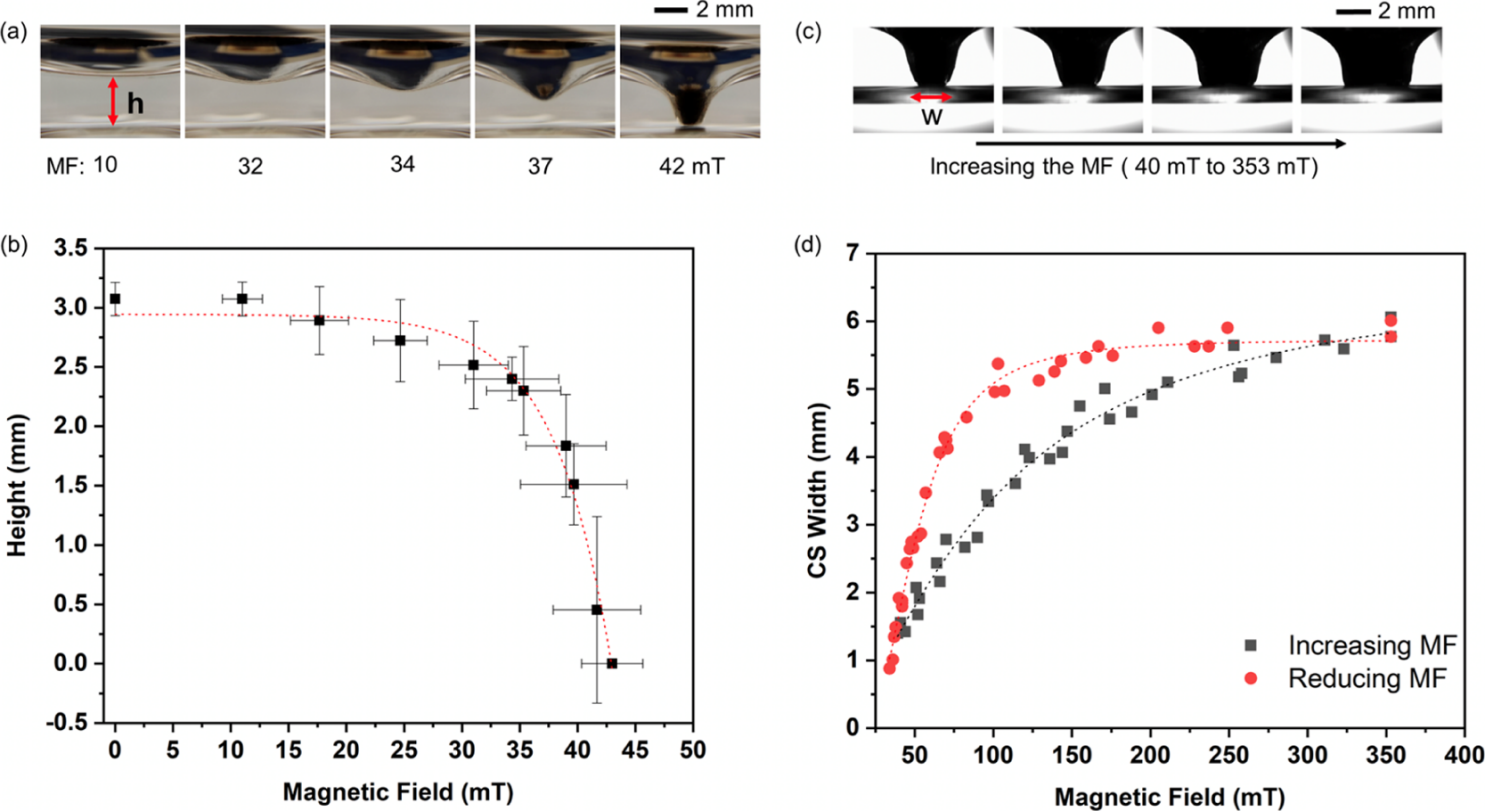
(a) Deformation of the SMNPs–water interface under the applied MF. (b) Decay of the distance between the (4 mg) SMNPs–water interface and the bottom of the glass vial of 5 mL (1 mL water volume), *n* = 3. (c) Pictures of the CS at different MFs (over 42 mT) and (d) hysteresis curve of the CS width values under an increasing (gray) and a reduced (red) MF.

The floating superhydrophobic magnetic NP colloid layer incurves downward forming a water–solid/water–air interface under the MF, and the observed bending is more pronounced with the increase of the MF value, as shown in Video S6.2. This particle-confined water interface deformation behavior is higher than the Moses effect suffered by a diamagnetic liquid under a MF.^24,28^ As presented in Figure 1, the solid–water interface touched the depth of the water container with the increment of the MF, making a stable flipped conical-structured water interface, with a twister-like shape. However, it was appreciated that the SMNPs exhibited two states over the water surface: as a monolayer of particles, attracted to the water interface under capillary forces, and as free SMNPs on top of the attracted monolayer at the end of the cone. Free SMNPs accumulated on the water interface conical spike region due to the magnetic field gradient. This increased particle density increased the magnetic moment and inflicted an inclination of the magnetic force in the middle of the conical shape, modifying the structure and making it more concave. The variation of the distance (*h*) between the water-superparamagnetic superhydrophobic NP (4 mg) interface and the floor of the glass vial, with the applied MF, is depicted in Figure 2a. A rapid increment of the conical shape spike was observed over ∼30 mT, along with an abrupt decaying of the *h* value, as shown in Figure 2b. Finally, at ∼42 mT, the spike touched the glass surface and generated a stable twister-shaped configuration. Then, the horizontal remote translocation of the twister was investigated by changing the position of the permanent magnet but keeping a constant MF (67 mT), as seen in Video S6.3.

When increasing the amount of magnetic NPs in the system, for instance, from 4 to 10 mg, a lower MF was needed for the twister to touch the bottom of the glass vial. This can be explained considering the increment of the magnetic moment in the whole system. Figure S4a shows that the required MF dropped to 31 mT when increasing the particle amount up to 10 mg. In the case of the magneto twister formation, the magnetic force, as shown in eq 1, acts as the main driving force for the origination of the magneto twister, and it depends on the amount of particles and the applied MF gradient, for the same type of particles and tested water levels. Therefore, the required gradient MF can be lowered (<31 mT, MF at the surface of the water container) when using more than 10 mg of SMNPs and can be raised (>42 mT, MF at the surface of the water container) when using less than 4 mg of SMNPs. Nevertheless, it needs to be considered that the required gradient MF is proportional to the height of the water layer at both low and high concentrations of particles.

An interesting observation was the width enlargement of the tip of the CS, once the twister was formed, by increasing the MF up to 353 mT, in both configurations (4 and 10 mg of SMNPs), as shown in Figure 2c. Most of the monolayer of SMNPs, which was spread over the water surface by capillarity, traveled to the bottom of CS of the twister when increasing the MF. Interestingly, they moved inclinable downward on the water surface without leaving the surface due to the lateral capillarity force, keeping the twister shape even during horizontal remote translocation, as shown in Video S6.3. In Video S6.1, it is possible to observe that the SMNPs traveled on the water surface under the horizontal (*x*-direction) gradient MF.

As explained above, the accumulation of SMNPs at the bottom surface of the vial brought an increase in the surface area of the CS of the twister and an enlargement of the width of the spike, as depicted in Figure 2c. Therefore, the forward and reverse manipulations of the CS neck of the twister were possible by increasing or decreasing the strength of the MF, as shown in Figure 2d. Interestingly, a significant hysteresis on the width value of the CS was observed when comparing the increasing and reducing MF paths. This behavior can be explained considering the adhesion forces between the SMNPs and the bottom of the glass vial, affecting the release of the SMNPs back to the water surface interface. Moreover, the magnetic dipole–dipole interaction could also affect the release process by keeping the particles in an aggregation state at high MFs. However, the buoyancy and the upward surface tension forces start to dominate the movement of the SMNPs back to the previous shape once reducing the MF on the whole system. A higher amount of magnetic SMNPs (10 mg) led to an increase in the size of the width of the CS, but it showed the same hysteresis trend, as shown in Figure S4b. Furthermore, the amount of SMNPs directly affects the enlargement of the width in the CS while increasing the MF, after the formation of the magneto twister. In this case, a lower saturation width (<6.0 mm) and a higher saturation width (>8.2 mm) are expected when using less than 4 mg or more than 10 mg of SMNPs, respectively.

Further characterization of the twister was done, as shown in Figure S5, presenting a kind of visible air column attached to the superhydrophobic SMNPs when in water, called the plastron effect,^29,30^ which was observed in three different states. In state-1, at a low MF, a visible plastron caused the air attraction on the superhydrophobic SMNPs. The inhomogeneous SMNP layer enhanced the confinement of a thick and stable air layer between the SMNPs and the water surface. However, in state-2, the higher MF promoted a more compact SMNP layer while increasing the width of the CS, then incrementing the pressure between the SMNP layer and the water interface, and consequently hindering the visibility of the air plastron; similar to the effect occurring in a Cassie-Baxter to Wenzel transition.^31^ Finally, in state-3, at even higher MFs, the attraction of the SMNPs to the external magnet along with their high magnetic flux promoted the accumulation of a highly visible air plastron layer around the SMNPs agglomerated at the bottom of the CS of the twister.

**Figure 3:**
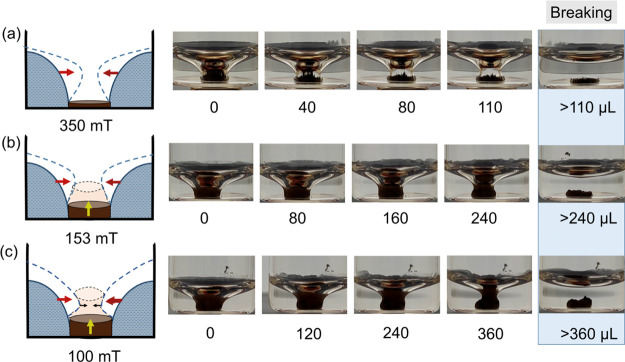
Characterization of the disruption of the CS shape during the addition of water to the twister under different MFs (a) 350, (b) 153, and (c) 100 mT.

The disorder of the twister shape was investigated by increasing the water column in the glass vial under different MFs, as seen in Figure 3. Incremental amounts of water volumes were added to the vial containing 1 mL of water. 4 mg of SMNPs was stable, maintaining the twister shape at 350, 153, and 100 mT, as seen in Figure 3. At high MFs, 350 mT, the water–air interface over the CS obtained by the SMNPs tried to stretch itself by repelling the superhydrophobic phase while adding the water until complete failure, resulting in the breaking of the twister. At 350 mT, the SMNPs got firmly captured at the surface of the glass vial, getting the water–air interface (left and right water–air interfaces in the 2D side view) closer to each other, as explained above. After an addition of 120 ± 12 μL of water, the water–air interface got fully disrupted and the SMNPs deposited at the bottom of the vial, capturing a bubble, thanks to the applied MF. Nevertheless, when lower MFs were applied, the SMNPs tended to move upward through the air–water interface due to both upward capillary and buoyancy forces promoted by the opposite lower MFs. In this case, the water intake threshold increased to 240 ± 20 μL in 153 mT and up to 360 ± 20 μL for 100 mT. At low MFs, the capillary and electrostatic attraction of the SMNP layer on the water interface stretched vertically, reducing the CS diameter, in a more drastic way, when increasing the volume of water, in comparison to the high MF experiments. As shown in Figure 3c at MFs of 100 mT, the water–SMNP layer interface was able to get closer to each other, generating narrower cones, while increasing the volume of water in the vial without breaking the twister shape. This effect can be explained considering that lower magnetic forces affected the SMNP layer at the top of the twister to a less extent, leaving a more homogeneous water–SMNP layer interface and generating stable twister shapes, even when increasing the volume of water in the vial.

### 2.2 Water Droplet Transport on an Aqueous Surface

**Figure 4:**
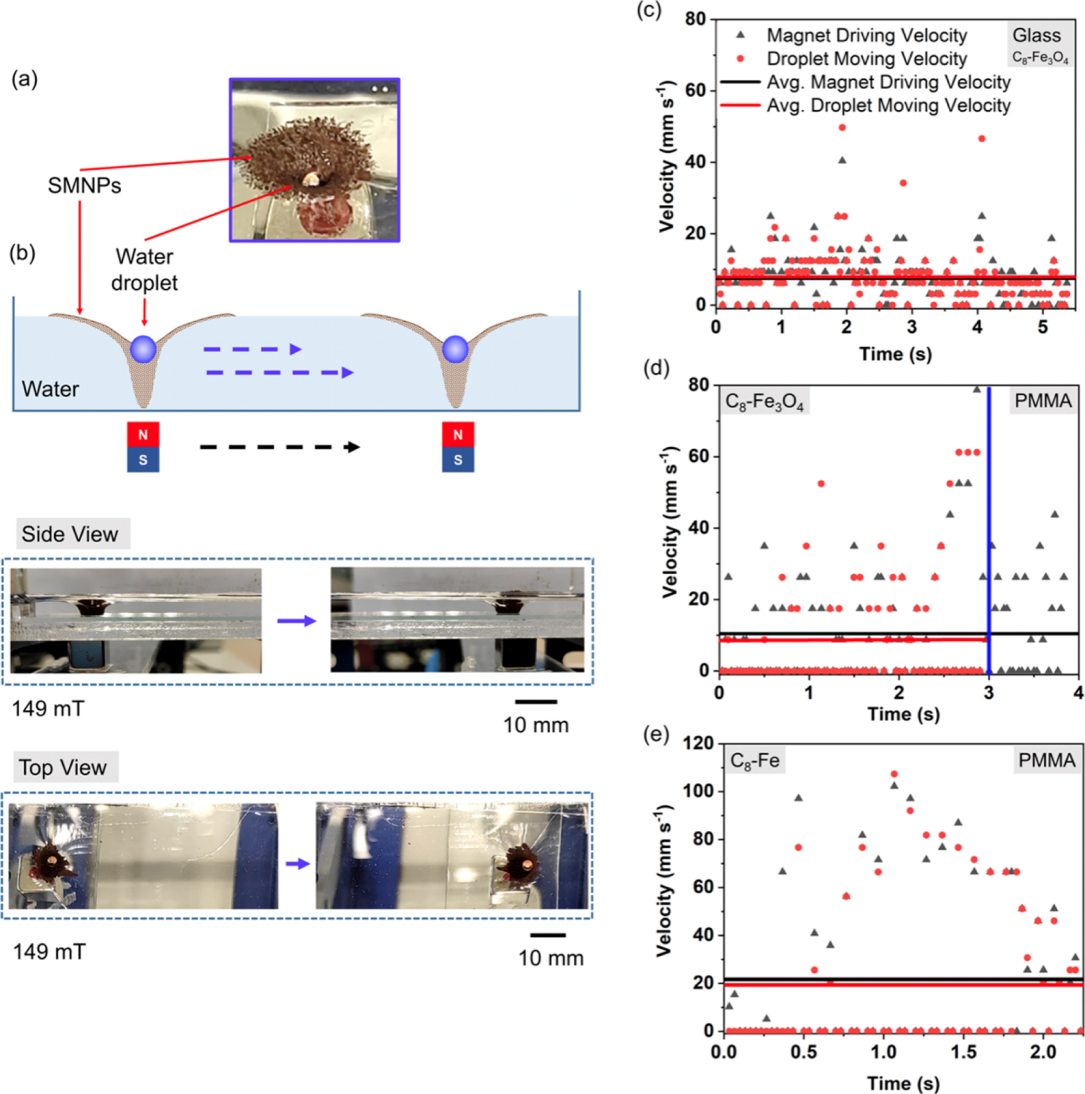
(a) Image of the twister carrying a water droplet of 5 μL (top view). (b) Schematic diagram of the water droplet movement on the water surface under the displacement of an applied gradient MF. Side view of the movement of a water droplet over the twister in an aqueous environment under 149 mT. Top view of the movement of a water droplet over the twister in an aqueous environment under 149 mT (horizontal translocation of the *z* direction MF). Velocity profiles for the movement of the droplet (red dots) and the permanent magnet (black triangles) by using the SMNPs twister for (c) a glass surface, (d) a PMMA surface, and (e) by using the superhydrophobic Fe particle twister for a PMMA surface in water.

In digital microfluidics, the remote-stimuli movement of microdroplets plays an important role by handling small volumes of liquids. For instance, Zhao et al.^13^ reported the manipulation of a magnetic liquid marble, a liquid droplet encapsulated by superhydrophobic and magnetic Fe_3_O_4_ NPs, to handle discrete volumes of liquids on a solid surface.^32^ Both magnetic liquid marbles and the introduced magneto twister operate by water–air interfaces adhered with superhydrophobic magnetic NPs, while an extra amount of energy is utilized in the magneto twister formation. However, the MF-induced formation of the magneto twister system facilitates the bending of the water–air interface adjacent to the magnetic particle–water interface by introducing a flipped conical structure, separating water–air interfaces beside the magneto twister. This twister formation facilitates additional characteristics such as the bending of the water–air interface and the tunability of the dimensional parameters of the twister and the substrate solid magneto twister surface area. These improve the applicability of the twister in both bulk water phases and discrete water droplets, when compared to magnetic liquid marbles. Moreover, for magnetic droplet manipulation, the magnetic liquid marbles show an unstable behavior while transporting the marbles due to friction and handling forces. In this regard, Khaw et al. reported a magnetically actuated floating liquid marble on a water surface to transport the droplet with a minimum friction force, when compared to solid surfaces.^33^ However, careful handling is required to manipulate magnetic liquid marbles. For instance, the magnetic shell easily opens with tiny external forces resulting in the mix of the droplet contents with the bulk liquid and smashing the droplet manipulation system. In this regard, our twister system can improve the manipulation of liquid droplets over the water surfaces without breaking the system under the unexpected handling forces. The twister structure was able to hold a water droplet over the CS without leaking or losing its volume over time, as seen in Figure 4a. The droplet was stabilized on the surface of the SMNP layer and could be transported through the surface using the movement of the external permanent magnet, as illustrated in Figure 4b. Videos S6.4 (top view) and S6.5 (side view) show the transport of a water droplet in a water environment while moving the droplet under a 149 mT MF. At low MFs (<42 mT, for this particular system), the water droplet sat on the CS of the twister, which did not touch the surface of the water container. In this case, zero friction between the spike and the water surface was anticipated. However, at this state, the whole system presented low stability while moving the permanent magnet, and the droplet easily collapsed to the bulk aqueous system. Although, at higher MFs (>118 mT, for this particular system), the water droplet was stable moving over the water surface due to the robust and well-packed SMNP layer under the MF. In contrast, the friction between the CS of the twister and the surface of the water container surface was found to be high, affecting the droplet movement performance. It was observed that the movement of the droplet was regulated by the adhesion force between the SMNP layer and the container surface. In order for the system to be useful for droplet manipulation, the effect on the translocation of a water droplet was investigated for two different surfaces, a glass (hydrophilic) and a polymethyl methacrylate (PMMA) (less hydrophilic) surface. The moving velocity of the twister structure carrying a water droplet and the driving velocity of the permanent magnet during the translocation process were monitored. When using the glass surface, as seen in Figure 4c, nearly the same average magnet driving (7.64 mm s^–1^) and droplet moving (7.91 mm s^–1^) velocities were observed, showing low friction due to the low adhesion force between the glass surface (∼58.40 mJ m^–2^, surface energy) and the low surface energy of the SMNP layer of the twister. On the other hand, when using a PMMA surface, the droplet was moved along the surface, but at a certain moment the droplet collapsed and mixed with the bulk, as shown in the blue vertical line in Figure 4d. The high surface adhesion force between the SMNP layer and the PMMA surface (surface energy ∼41.21 mJ m^–2^) resulted in the deposition of the NPs coming from the CS on the PMMA surface, destroying the SMNP layer structure during the movement of the twister.

However, this behavior was avoided by using superhydrophobic Fe particles, which have a higher saturation magnetization, 220 A m^2^ Kg^–1^ (3.9 times higher magnetization than SMNPs), as shown in Figure 4e. The higher magnetic forces of these particles were attracted more firmly to the moving magnet and overcame the high adhesion force between the particles and the PMMA surface, recovering a stable droplet manipulation system. Therefore, the stable droplet manipulation in water was possible by manipulating the surface energy, the magnetic force applied, and the chemistry of the particle system forming the twister. This has specific implications on the efficient transportation of aqueous-based reactive droplets inside of an aqueous medium in a controllable way in the same compartment without merging two miscible aqueous liquid phases. These findings could provide a solution to reduce the instability of remotely stimulated magnetic liquid marbles.

### 2.3 Magneto Controllable Plugs

**Figure 5:**
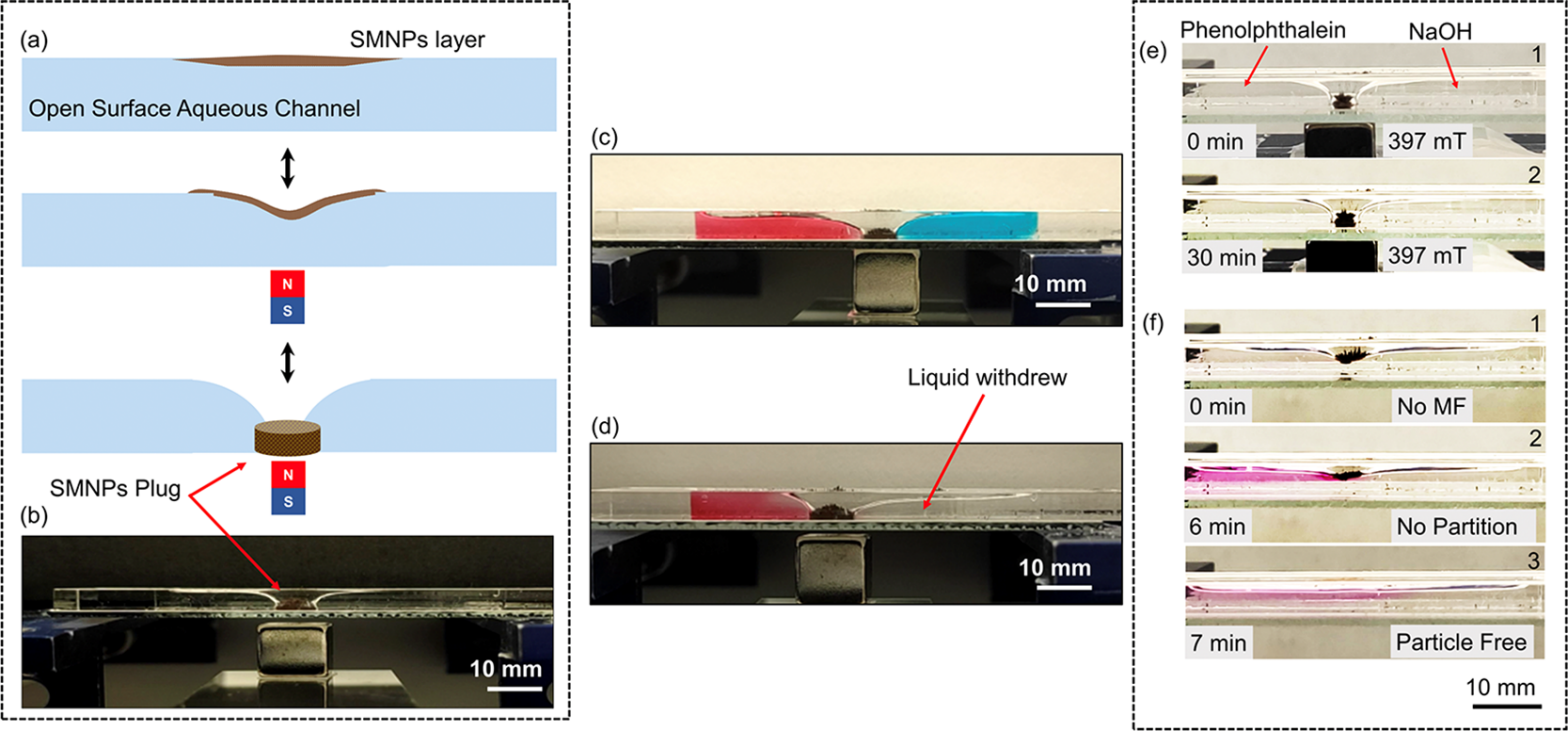
(a) Schematic illustration of the formation of the plug. (b) Picture of the magnetic plug in an open surface PMMA channel. (c) Picture showing two different color liquids on both sides of the plug, demonstrating the absence of leaking or diffusion after partition. (d) Picture of the open channel after withdrawing some liquid from the right side partition. (e) Pictures of the partition of phenolphthalein (pH indicator) and NaOH solutions by the magnetic plug. (f) Pictures of the removal of the plug by removing the MF, observing the mixing of the two partitions, visually indicated by the formation of the purple/pink color of the phenolphthalein in alkaline media.

Remotely controllable plugs and valves are promising tools to be applied in the lab-on-chip technology. In this regard, magnetic particles with different functionalities have been used as plugs to analyze biomarkers,^34,35^ magnetic particle-impregnated hydrogels as valves^36^ and plugs,^37^ and magnetic particle-stabilized liquids (ferrofluids) as valves to change the direction of flow.^38^ Interestingly, the deformation of the superhydrophobic magnetic particle–water interface system can be easily applied for the generation of plugs in open channels, as seen in the scheme in Figure 5a. The water–SMNP interface deformed to form the twister in the middle of the open channel under an applied MF. By increasing the value of the MF, the SMNPs were compacted at the bottom of the channel, keeping the twister structure and thus forming a superhydrophobic plug, separating the liquid into two independent partitions, as shown in the picture in Figure 5b. The switching off of the MF allowed the release of the particles from the bottom of the channel, bringing them back to the water surface, and removing the magnetic plug. Liquid separation was achieved in the channel by the formation of an elongated meniscus attached to the SMNPs. In another experiment, as depicted in Figure 5c, two different liquids (red and blue) were independently stored by the plug, avoiding liquid leakages, mixing, or diffusion. Moreover, the stability of the plug was investigated in order to demonstrate that it was possible to reduce the volume in one of the containers, acting as an independent liquid reservoir. By removing the liquid from one of the reservoirs, the formation of a non-equivalent meniscus level in both sides of the plug demonstrated the sealing capacity of the plug under the MF. The liquid in the right side partition (colorless) of the plug was successfully removed (∼30% of its volume) by creating a negative flow pressure on the right side of the channel, as seen in Figure 5d. No diffusion was observed for the red color liquid, from the left to the right side of the plug.

Moreover, to further demonstrate the perfect separation of the water partitions, a NaOH solution of pH ∼ 12.5 and colorless and a solution containing phenolphthalein of pH ∼ 6–7 and colorless, were separated by the twister structure, as seen in Figure 5e. After 30 min, no purple/pink color (color of the phenolphthalein in basic media) was observed in the surroundings of the partition, demonstrating that the twister prevented the leaking or diffusion of the liquids in the partitions. Moreover, the removal of the MF facilitated the mix of the two solutions when releasing the plug, allowing the formation of the purple/pink color in less than 5 min, as seen in Figure 5f, see Video S6.6. This allows the use of this type of switchable plug for the formation of independent reaction chambers in open channels, which can be independently manipulated and transformed. The observed effect has important applications in microfluidic devices.

### 2.4 Removal of Floating Microplastics Using the Twister from the Water Interface

**Figure 6:**
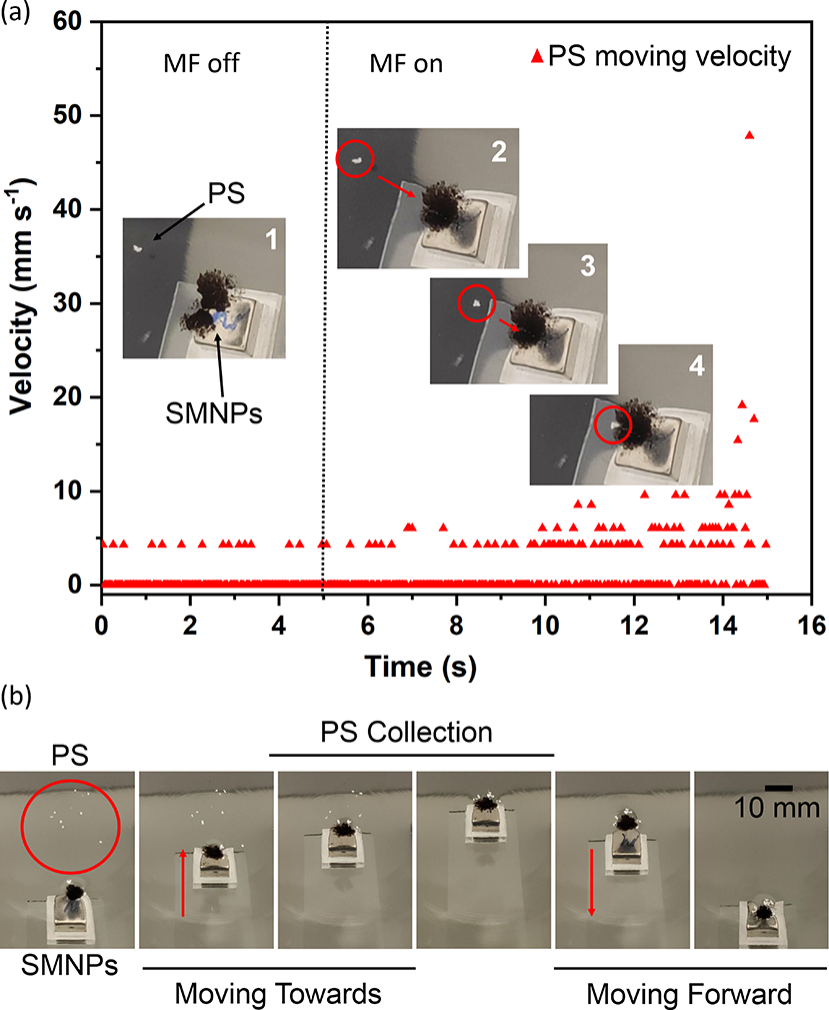
(a) Velocity of a PS particle attracted toward the twister, without a MF applied to the SMNP layer (before 5 s time, picture 1) and with a MF of 100 mT, (after 5 s time, pictures 2–4). (b) Set of images taken from Video S6.7. The water surface was “contaminated” with PS microparticles, then the twister was moved toward the PS microparticles and removed them from the surface.

The presence and accumulation of floating microplastics in natural waters is a harmful situation that leads to the pollution of water resources.^39^ Therefore, treating and manipulating contaminated water–air interfaces is urgent and needs to be properly addressed. Microplastic contamination is expected in miniaturized devices as well, coming from the devices’ fabrication processes; thus, cleaning them by a remote stimulus will provide no additional steps to the process. In this regard, magneto manipulation of the water–air interface deformation by the SMNP layer could be applied to collect and remove microplastics from the water surface. In order to prove this assumption, PS particles sprinkled over the water surface of a half-filled glass Petri dish, were used as a proof of concept of a “contaminated” water surface. Then, 4 mg of SMNPs was sprinkled at 12.5 mm far from the PS particles. It was observed that the SMNPs did not attract the PS particles. The PS particles remained static without moving toward the SMNPs layer, keeping their velocity nearly 0 mm s^–1^, as shown in Figure 6a (with the MF off). After 5 s, the magnet was brought near the SMNP layer (100 mT), forming the twister shape structure (with the MF on), as explained above, activating the water interface deformation, CS, as seen in Figure 1. At that moment, a PS microparticle started to move toward the twister (see the pictures in Figure 6a), starting to accelerate toward the twister, and achieving a maximum speed of 47.8 mm s^–1^ just before being adsorbed by the CS. The trajectory of the PS particle is shown in Video S6.7, in real time, starting with slow and random movements, in which the velocity increased when approaching the center of the CS. The twister formed a steep water–air interface, which provided a short of slide configuration for the particle to move toward the twister. Moreover, the lateral capillary forces and the van der Waals forces between the SMNPs and the PS microparticle acted as interacting forces, accelerating the movement of the particle.^40^

Considering this finding, the translocation of the twister, using the external MF, was used to capture the non-magnetic PS particles present at the surface of the water by just moving the permanent magnet and thus the twister. Figure 6b shows a set of images, taken from Video S6.8, of the movement of the twister, capturing multiple PS microparticles. By moving the twister around the water surface, all the PS microparticles can be easily collected and removed since they hold on to the SMNPs of the twister by the lateral capillarity and van der Waals forces while displacing the magnetic twister. Therefore, this novel strategy provides an easy and cheap technique to remove water surface-contaminated plastic particles by using the magnetic twister.

## 3 Conclusions

In conclusion, a novel low-surface-energy superhydrophobic magnetic particle system is introduced, called “magneto twister”, which is a stable, magneto-tunable and movable, flipped, conical-structured, solid–water interface, resembling a storm twister in shape. The magneto twister was fully characterized to elucidate the properties and limitations of the system. The applicability of this system was reported for three different potential microtechnological applications. First, the magneto twister was used to manipulate water droplets in a water environment by just placing a water droplet over the twister and transporting the droplet by displacing the applied MF, facilitating water droplet translocation in aqueous media. Due to the robustness of the magneto twister, the droplets were manipulated in a stable manner, which is a problem commonly faced in magnetic liquid marble particle systems. Further, the twister was introduced as a magnetic plug to separate liquids inside of an open surface channel. Finally, the magneto twister was used to collect and remove floating microplastic particles from the surface of the water by simply moving the twister toward the microplastic to trap and then remove them by magnetic guiding. This investigation opens up new pathways for using superhydrophobic magnetic NPs in water–air interface-assisted applications.

## 4 Experimental Section

### 4.1 Synthesis of Superhydrophobic Magnetic Particles

First, 0.5 M ferric solution was prepared by dissolving 2.71 g of FeCl_3_·6H_2_O (>99%, Sigma-Aldrich, Spain) in 20 mL of distilled water and then 0.5 M ferrous solution was prepared by dissolving 1.39 g of FeSO_4_·7H_2_O (>99%, Sigma-Aldrich, Spain) in 10 mL of distilled water. Then, 30 mL of Fe^3+^/Fe^2+^ (Fe^3+^/Fe^2+^, 2:1) solution (deoxygenated) was added drop-by-drop into 40 mL of 1 M NaOH (>98%, Sigma-Aldrich, Spain) solution, which was then kept at 40 °C under vigorous stirring in a N_2_ atmosphere.^41^ A black precipitate was immediately formed. Then, the precipitate was heated at 90 °C for 30 min. After that, the precipitate was separated from the solution with a magnet and washed three times with distilled water. Finally, the Fe_3_O_4_ NPs were dried by rotary evaporation at 40 °C under vacuum. Next, 1.8 mL of triethoxy(octyl)silane (97%, Sigma-Aldrich, Spain) was mixed with 50 mL of absolute ethanol (Sharlau, Spain) for 2 h at 50 °C. Then, a sonicated suspension of pre-synthesized 0.53 g of Fe_3_O_4_ NPs in 10 mL of absolute ethanol was added to the triethoxy(octyl)silane/ethanol solution and stirred for 2 h. Later, the particles were separated with a magnet and thoroughly washed with ethanol three times.^42^ Finally, the particles were heated at 120 °C for 2 h to obtain SMNPs.

### 4.2 Magneto Deformation of the SMNP-Confined Water–Solid–Air Interface

4 mg of the synthesized SMNPs was sprinkled on the water surface (1 mL of water in a glass vial of 5 mL). The gradient MF was supplied by changing the vertical distance (*z*) between the depth bottom of the glass vial and the NdFeB permanent magnet (cubic 10 mm magnet, 495 mT). The MF (*B*) was calculated by eq 2,^33^ where *B*_r_ is the remanence field, *L* is the length, *W* is the width, *D* is the height of the magnet, and *z* is the distance from the magnet surface.
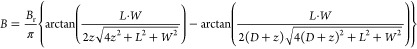
2

The breaking of the CS was investigated by adding water to the glass vial at fixed MFs: 350, 153, and 100 mT.

### 4.3 Water Droplet Transport on Aqueous Media

A water droplet of 5 μL was placed inside of the CS, formed by 10 mg of SMNPs in a container with a water height of 6.3 ± 0.5 mm. The magnet was manually moved horizontally to shift the position of the CS together with the water droplet. The experiments were carried out using glass and PMMA substrate containers. Superhydrophobic Fe particles (commercially available Fe microparticles (99.9% Thermo Fisher, Spain) were treated with dilute HCl (0.25 M) for 10 min and coated with triethoxy(octyl)silane following the same protocol mentioned in Section 2.1, and they were also used to form the CS and water droplet transport experiments. The moving velocities of the magnet and of the magneto twister, with the water droplet, were tracked by video analysis, through the ImageJ software coupled with the manual tracking plugin. This was used to track the motion of the magneto twister and the magnet within short time ranges, which allowed one to obtain velocity values.

### 4.4 Magneto Controllable Plug Formation

For the plug experiments, a 4 × 2 mm (height/width) open surface channel of 5 cm length, made of polymethylmethacrylate (PMMA), Goodfellow, Spain, with a glass microscope slide as the bottom surface, bound together by a 380 μm double-side pressure-sensitive adhesive (PSA) layer (Adhesive Research, Ireland) was used. The PMMA layer was fabricated using a CO_2_ laser system (VLS2.30 Desktop Universal Laser System) equipped with a 10.6 μm CO_2_ laser source ranging in power from 10 to 30 W.

The channel was filled with 350 μL of water and then 2 mg of SMNPs was sprinkled on the open surface water interface. Finally, the magnetic plug was constructed by using the permanent 1 mm cubic magnet (∼397 mT). The two liquid partitions were colored using a water-based dye to demonstrate the absence of leaking from the plug. The liquid was withdrawn using a pipet into one of the partitions to check the effect of the SMNPs plug.

In order to check whether there was any leaking when using the plug, 60 μL of 31 mM phenolphthalein water solution and 60 μL of 1 M NaOH solution were added to the two generated compartments.

### 4.5 Removal of Floating Microplastics from the Water Interface

Polystyrene (PS) microparticles (diameter, 0.5–1.0 mm) were suspended in a half-filled glass Petri dish (*h* = 8.0 ± 0.5 mm), and 4 mg of SMNPs was sprinkled on the water surface. The twister shape was formed under the MF (105 mT), and the permanent magnet was moved toward the PS particles. The PS particles were attracted to the twister shape and get collected. The twister shape was moved under a fixed MF over the surface of the Petri dish to collect the PS particles.

### 4.6 Characterization

TEM images of Fe_3_O_4_ NPs (in water suspension) were collected from a JEOL JEM 1400 Plus (JEOL, Japan). FTIR spectrum analysis of the NPs were carried out, in the transmittance mode, using a Jasco 4200 spectrometer. The Raman spectra were recorded by a Renishaw InVia Raman spectrometer, connected to a Leica DMLM microscope. The spectra were acquired with a Leica 50× N Plan (0.75 aperture) objective and a 785 nm laser (diode laser, Toptica). XRD patterns were collected by using a Philips X’pert PRO automatic diffractometer operating at 40 kV and 40 mA, in the theta–theta configuration, equipped with a secondary monochromator with Cu Kα radiation (λ = 1.5418 Å) and a PIXcel solid-state detector (active length in 2θ 3.347°). Data were collected from 6 to 80° 2θ, with a step size of 0.026° and time per step of 450 s at RT (scan speed, 0.015° s^–1^). Magnetization measurements for the iron oxide NPs and the dehydrated beads were carried out in a 5 T Quantum Design MPMS3 (SQUID) magnetometer at room temperature.

A layer of particles was coated onto a PSA tape to obtain the contact angle of the particle layer. Contact angles were measured using a DataPhysics OCA 15 EC drop shape analyzer, and the surface energies were obtained by the Owens, Wendt, Rabel, and Kaelble (OWRK) model. The images and the videos were recorded with a Sony IMX586 Exmor RS 48 megapixel lens with a 12× macro lens. The videos and the images were analyzed using the ImageJ software, and the particle tracking was done by the manual tracking ImageJ plugin.

## Supporting Information Available

Synthesis and characterization of superhydrophobic magnetic particles, XRD and Raman characterizations, TEM characterization, 10 mg SMNPs CS characterization, and images of the different states of the magneto twister (PDF)
Superhydrophobic magnetic particles on a water surface (MP4)
Formation of the CS under a MF (MP4)
Manipulation of the spike (MP4)
Top view of water droplet transport in aqueous medium (MP4)
Side view of water droplet transport in aqueous medium (MP4)
Magneto controlable plug (MP4) Trajectory of PS particles (MP4)
Removal of PS particles from water (MP4)
