# Supplementary material for: Magneto Twister: Magneto Deformation of the Water–Air Interface by a Superhydrophobic Magnetic Nanoparticle Layer
Source: Langmuir. 2022 Mar 9;38(11):3360–9. doi: 10.1021/acs.langmuir.1c02925 (PMC8945397; doi:10.1021/acs.langmuir.1c02925)
Supplement: Supplementary file 1 — la1c02925_si_001.pdf [file la1c02925_si_001.pdf]

Supplementary Information

Magneto Twister: Magneto deformation of water-air interface by a superhydrophobic magnetic nanoparticles layer

Udara Bimendra Gunatilake[a,b], Rafael Morales,[c,d] Lourdes Basabe-Desmonts[b,d,e,f*], Fernando Benito-Lopez,[a,e,f,*]

[a] Microfluidics Cluster UPV/EHU, Analytical Microsystems & Materials for Lab-on-a-Chip (AMMa-LOAC) Group, Analytical Chemistry Department, University of the Basque Country UPV/EHU, Spain.

[b] Microfluidics Cluster UPV/EHU, BIOMICs microfluidics Group, Lascaray Research Center, University of the Basque Country UPV/EHU, Vitoria-Gasteiz, Spain

[c] Department of Physical-Chemistry and BCMaterials, University of the Basque Country UPV/EHU, 48940 Leioa, Spain

[d] Basque Foundation of Science, IKERBASQUE, María Díaz Haroko Kalea, 3, 48013 Bilbao, Spain

[e] Bioaraba Health Research Institute, Microfluidics Cluster UPV/EHU, Vitoria-Gasteiz, Spain

[f] BCMaterials, Basque Center for Materials, Applications and Nanostructures, UPV/EHU Science Park, Leioa, Spain

SI-1 Synthesis and characterization of the superhydrophobic magnetic particles

The Fe_3O_4 nanoparticles were synthesized by the co-precipitation of ferrous and ferric ions under high pH conditions according to reaction-1.

$$2FeCl_3 + FeSO_4 + 8NaOH \rightarrow Fe_3O_4\ (NP) + 6NaCl + Na_2SO_4 + 4H_2O \quad \text{reaction-1}$$

The surface energy of the synthesized nanoparticles was reduced by chemically coating the Fe_3O_4 NPs with triethoxy(octyl)silane, as mentioned below. The possible chemical structure of the SMNPs is illustrated in SI-Figure 1a, top.

The FTIR spectra of both modified SMNPs and non-modified NPs were analyzed in order to investigate the binding of the alkyl chain *via* silane group, SI-Figure 1b. The C-H stretching vibrational peaks at 2850, 2900, 2960 cm^{-1} and Si-CH alkyl group bending vibrational peak at 1457 cm^{-1} in SMNPs FTIR spectrum, confirmed the presence of the alkyl chain within the SMNPs.[1] Moreover, the stretching vibrational peaks at 860, 990 cm^{-1} in the SMNPs spectrum were assigned to Si-C, Si-O-Fe bonds respectively, demonstrating the binding of the alkyl chain through the silane group to the NP surface.[1–3] The common peaks of Fe-O stretching, residual water OH stretching and bending were found in both modified and non-modified NPs at the positions of 576/580 cm^{-1}, 3425/3400 cm^{-1} and 1620 cm^{-1}.[1] However, the intensity of the O-H peaks was reduced in the SMNPs spectrum due to the hydrophobic alkyl groups, inhibiting the absorption of residual water to the surface of the NP.

The wetting properties of both types of the particles were characterized by the static contact angles (CA) of the particle layers, SI-Figure 1a, bottom. Bare NPs exhibited a superhydrophilic nature, by instantly absorbing the water droplet, showing nearly 0 ° water contact angle. Surface absorbed hydroxyl groups possess a high affinity to interact with water due to polar-polar attractions between water and pristine magnetic nanoparticles. However, the alkyl chain modified particles, exhibited superhydrophobic properties by showing a ~ 152.0 ° static water contact angle with a low surface energy, 11.85 mJ m^{-2}. The non-polar octyl-alkyl long hydrocarbon chain repels water molecules by the non-favourable attraction forces occurring between the carbon long chain and water molecules.

Then, the iron oxide phase of both SMNPs and non-modified NPs were characterized by the XRD and Raman spectroscopy, see section SI-2. Maghemite phase contamination was found in the magnetite phase, in triethoxy(octyl)silane coated nanoparticles, due to the

heat treatment at the coating process. In addition, a non-perfect spherical shape was obtained in both NPs types with 10.8 nm and 9.1 nm particle sizes, respectively, see SI-3 in supplement information.

The magnetic properties of both types of NPs were characterized with the SQUID magnetometer as shown in the SI-Figure 1c. The chemical modification on Fe_3O_4 NPs cost to drop the saturation magnetization down to 57 Am^2 Kg^{-1} from 62 Am^2 Kg^{-1}. Both types of NPs exhibited closely superparamagnetic behavior at room temperature, but SMNPs showed lower coercivity and remanence values compared to bare NPs, due to their molecular coating, which reduced the aggregation of the NPs, reduce the deviation from the ideal superparamagnetism. Nevertheless, the high saturation magnetization of the nanoparticles reflected the possibility to be used for remote manipulation under low (< 0.5 T) magnetic fields.

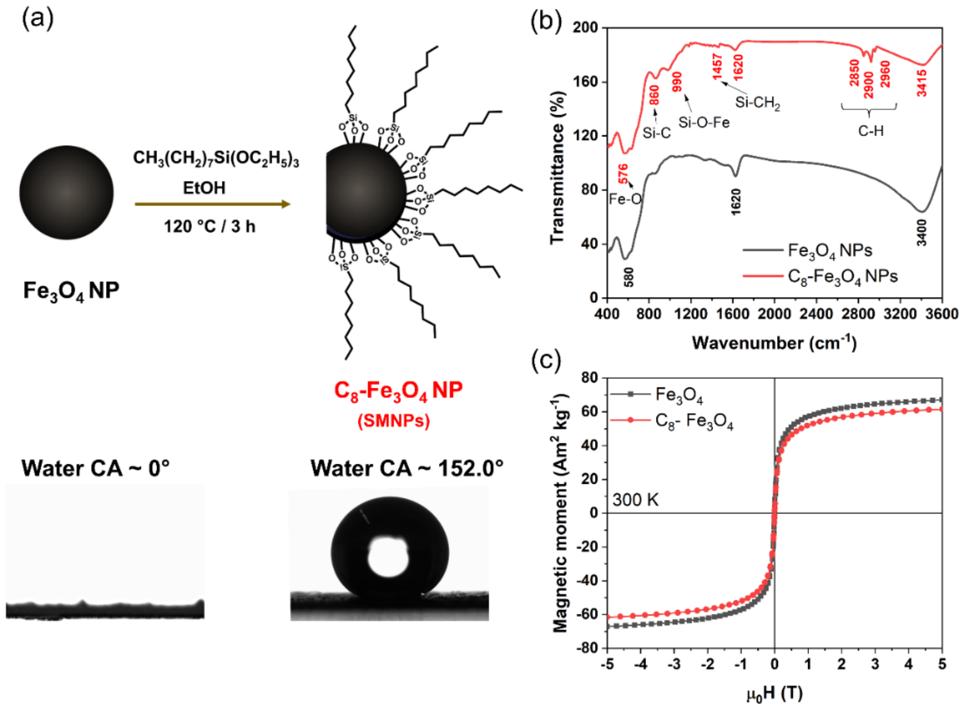

Figure SI-1: (a) Illustration of the chemical modification and the possible chemical structure of the modified SMNPs (C_8-Fe_3O_4), static water contact angles (CA) are shown under each type of particles (b) FTIR spectra and (c) SQUID analysis of the SMNPs (red) and non-modified NPs (black).

SI-2 XRD and Raman Characterisation

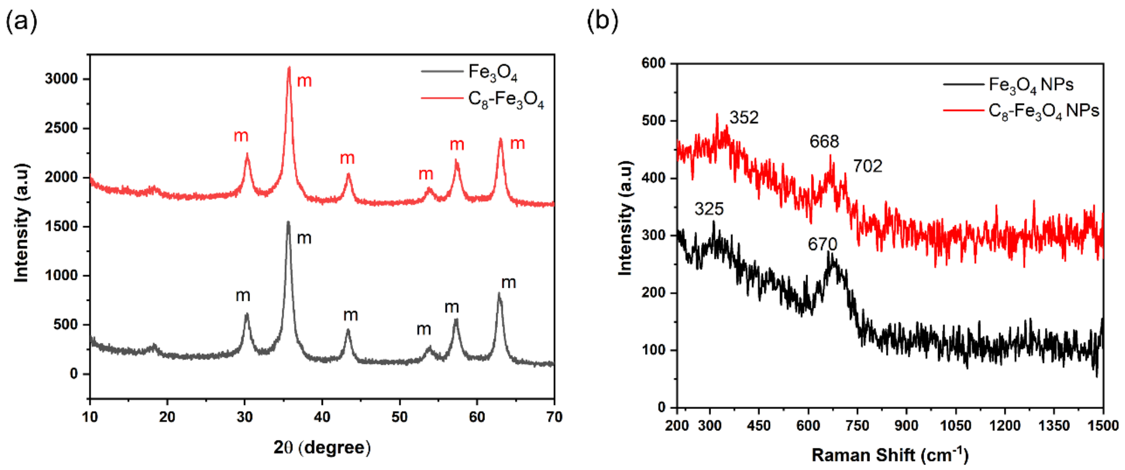

Figure SI-2: (a) XRD spectra (b) Raman spectra of the co-precipitated Fe_3O_4 nanoparticles and the chemically modified Fe_3O_4 nanoparticles (C_8-Fe_3O_4).

The phase of the synthesised iron oxide, both bare and the octyl alky chain coated iron oxide, was investigated by using XRD and Raman spectra. Conventional diffraction peaks related to the magnetite phase, were observed for both bare and octyl alky chain coated nanoparticles at 2θ = 30.3 °/30.4 °, 35.7 °/35.6 °, 43.4 °/43.4 °, 53.9 °/53.8 °, 57.5 °/57.4 °, and 63.0 °/63.0 ° respectively, (JCPDS 01-088-0315)[4] without showing any significant crystal deviation after triethoxy(octyl)silane coating, as depicted in Figure SI-1a. Further, Raman spectra were analysed for the both type of particles as shown in Figure SI-1b, to distinguish the magnetite and the maghemite phases since they were not distinguishable by XRD analysis, since they both have the same XRD diffraction angles. The Raman peaks at 670 (strong) and 610 (week) are attributed to the A_{1g} and T_{2g} Raman modes of the magnetite phase in Figure SI-1b, co-precipitated bare iron oxide nanoparticles. However, the magnetite A_{1g} mode split, and a peak at 702 (maghemite A_{1g} mode) appeared in the C_8-Fe_3O_4 NPs. Moreover, the 310 magnetite peak was red shifted to 352, reflecting the oxidation of magnetite to the maghemite in the C_8-Fe_3O_4 NPs, due to the heat treatment during the chemical modification process.[5] This reveals a small amount of maghemite contamination in the chemically modified Fe_3O_4 nanoparticles.

SI-3 TEM characterisation

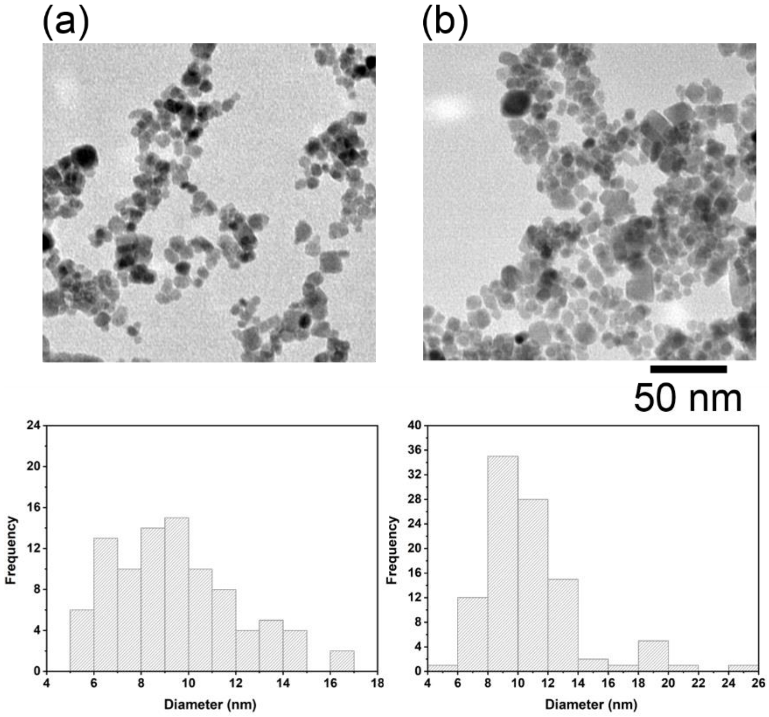

Figure SI-3: TEM images and particle size histograms of (a) co-precipitated Fe_3O_4 nanoparticles and (b) the chemically modified Fe_3O_4 nanoparticles.

SI-4 10 mg SMNPs conical spike characterization

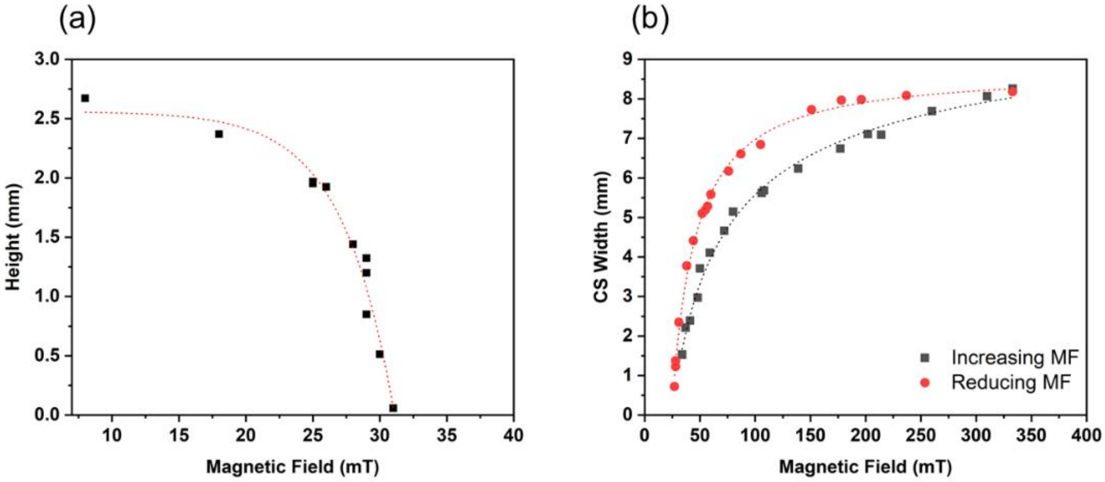

Figure SI-4: (a) Decay of the distance between the (10 mg) SMNPs-water interface and the bottom of the glass vial of 5 mL (1 mL water volume). (d) Hysteresis curve of the conical shape (CS) width values under an increasing (grey) and reduced (red) magnetic field (MF).

SI-5 Different states of the magneto twister

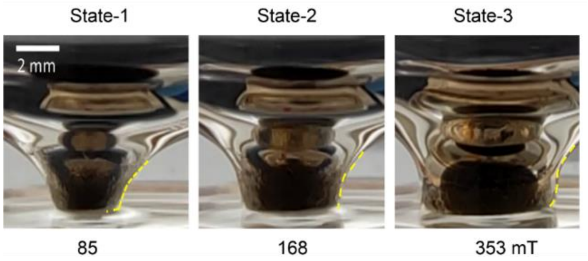

Figure SI-5: Visualization and characterization of the diffusion states of the air plastron in the CS of the twister, at different MFs. Broken yellow lines indicate the boundary of the water interface.

Videos SI-6

- Video SI-6.1: Superhydrophobic magnetic particles on water surface.
- Video SI-6.2: Formation of the conical spike under magnetic field.
- Video SI-6.3: Manipulation of the spike.
- Video SI-6.4: Water droplet transport in aqueous medium (top view).
- Video SI-6.5: Water droplet transport in aqueous medium (side view).
- Video SI-6.6: Magneto controlable plug.
- Video SI-6.7: Trajectory of the polystyrene particle.
- Video SI-6.8: Removal of polystyrene particle on water.

References

(1) Wang, J.; Meng, G.; Tao, K.; Feng, M.; Zhao, X.; Li, Z.; Xu, H.; Xia, D.; Lu, J. R. Immobilization of Lipases on Alkyl Silane Modified Magnetic Nanoparticles: Effect of Alkyl Chain Length on Enzyme Activity. *PLoS One* **2012**, *7* (8), e43478.

(2) Herth, E.; Zeggari, R.; Rauch, J. Y.; Remy-Martin, F.; Boireau, W. Investigation of Amorphous SiOx Layer on Gold Surface for Surface Plasmon Resonance Measurements. *Microelectron. Eng.* **2016**, *163*, 43–48.

(3) Devouge, S.; Conti, J.; Goldsztein, A.; Gosselin, E.; Brans, A.; Voué, M.; De Coninck, J.; Homblé, F.; Goormaghtigh, E.; Marchand-Brynaert, J. Surface Functionalization of Germanium ATR Devices for Use in FTIR-Biosensors. *J. Colloid Interface Sci.* **2009**, *332* (2), 408–415.

(4) Gates-Rector, S.; Blanton, T. The Powder Diffraction File: A Quality Materials

Characterization Database. *Powder Diffr.* **2019**, *34* (4), 352–360.

(5) Schwaminger, S. P.; Bauer, D.; Fraga-García, P.; Wagner, F. E.; Berensmeier, S. Oxidation of Magnetite Nanoparticles: Impact on Surface and Crystal Properties. *CrystEngComm* **2017**, *19* (2), 246–255.
